# Author Correction: Clopidogrel as a donor probe and thioenol derivatives as flexible promoieties for enabling H_2_S biomedicine

**DOI:** 10.1038/s41467-018-06832-8

**Published:** 2018-10-10

**Authors:** Yaoqiu Zhu, Elkin L. Romero, Xiaodong Ren, Angel J. Sanca, Congkuo Du, Cai Liu, Zubair A. Karim, Fatima Z. Alshbool, Fadi T. Khasawneh, Jiang Zhou, Dafang Zhong, Bin Geng

**Affiliations:** 1 Department of Chemistry and Biochemistry, Border Biomedical Research Center, The University of Texas at El Paso, El Paso, TX 79968 USA; 2 Hypertension Center, Fuwai Hospital, CAMS-PUMC, State Key Laboratory of Cardiovascular Disease, Beijing 102300, China; 3 State Key Laboratory of Drug Research, Shanghai Institute of Materia Medica, Chinese Academy of Sciences, 501 Haike Road, Shanghai 201203, China; 4 Department of Pharmaceutical Sciences, School of Pharmacy, The University of Texas at El Paso, El Paso, TX 79902 USA; 5 Analytical Instrumentation Center, College of Chemistry and Molecular Engineering, Peking University, Beijing 100871, China

Correction to: *Nature Communications* ; 10.1038/s41467-018-06373-0; published online: 27 September 2018

The original version of this article contained an error in the spelling of the author Bin Geng, which was incorrectly given as Bing Geng. This has been corrected in both the PDF and HTML versions of the article.

