# Supplementary material for: Clopidogrel as a donor probe and thioenol derivatives as flexible promoieties for enabling H2S biomedicine
Source: Nat Commun. 2018 Sep 27;9:3952. doi: 10.1038/s41467-018-06373-0 (PMC6160475; doi:10.1038/s41467-018-06373-0)
Supplement: Supplementary file 1 — Supplementary Information [file 41467_2018_6373_MOESM1_ESM.pdf]

Supplementary Information

for

Clopidogrel as a Donor Probe and Thioenol Derivatives as Flexible Promoieties for Enabling H_2S Biomedicine

Zhu *et al.*

Supplementary Figures

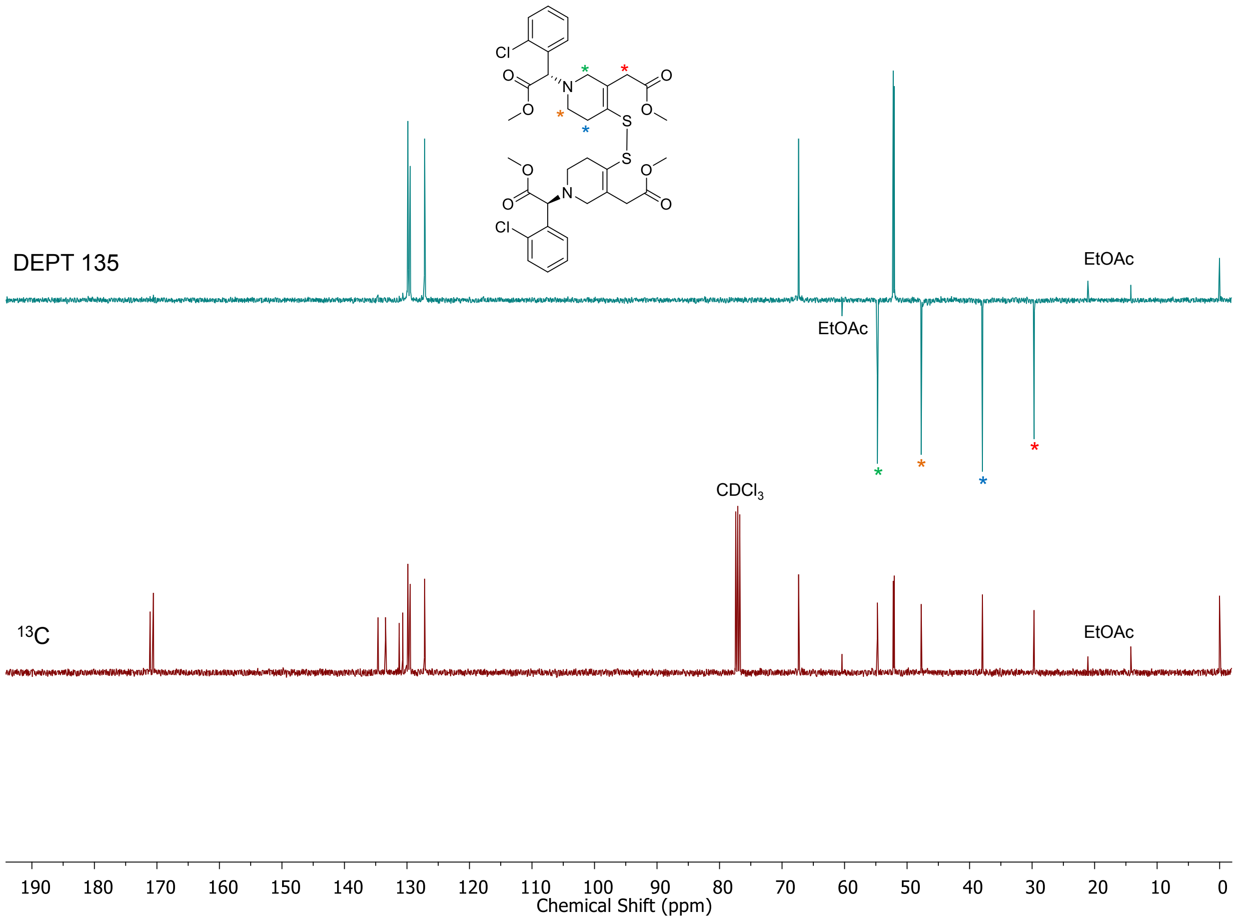

Supplementary Figure 1. NMR ($CDCl_3$, 100 MHz) of M15-OMe-DS. DEPT 135 spectrum supports the endo double bond structure of M15-OMe-DS.

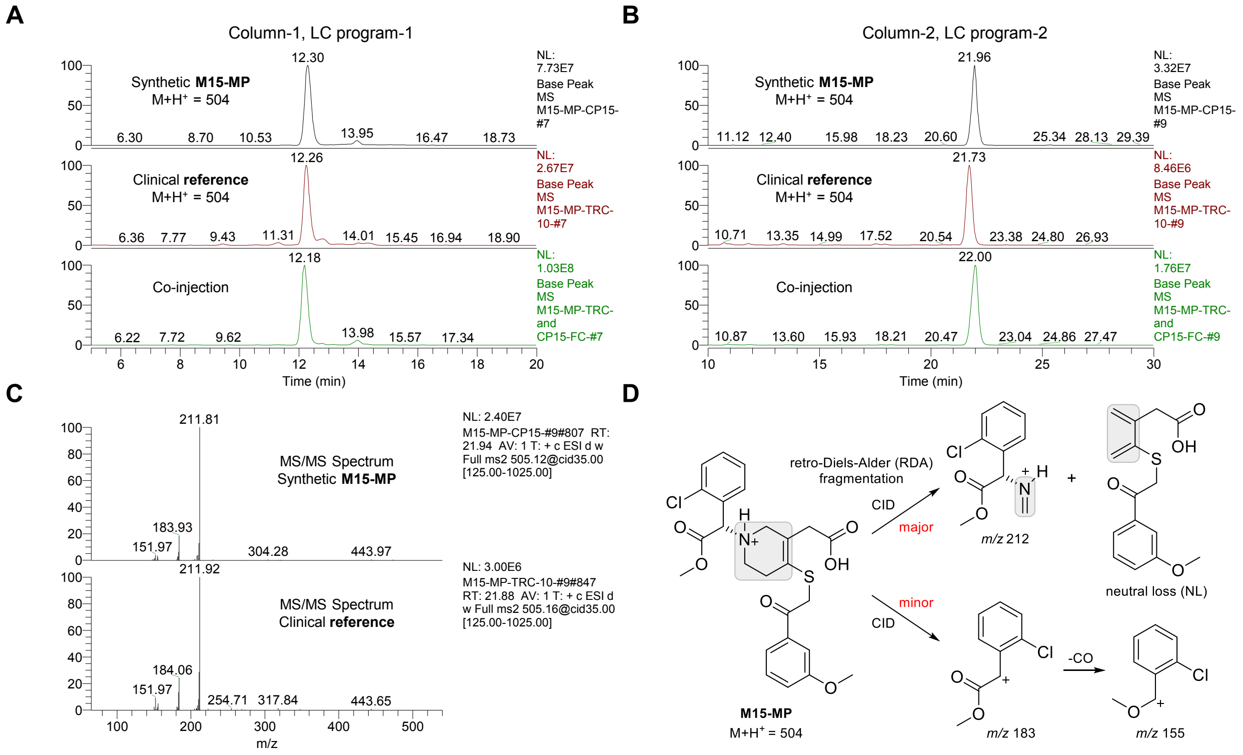

Supplementary Figure 2. Charaterization of synthetic M15-MP. (A) LC-MS/MS analysis on column-1 (Shimadzu TestKit, 50 x 4.6 mm, 5.0 µm) with LC program-1 (linear gradient of 10% to 95% B over 2-12 min). (B) LC-MS/MS analysis on column-2 (Agilent Zorbax C18, 150 x 4.6 mm, 5.0 µm) with LC program-2 (linear gradient of 10% to 95% B over 0-29 min). (C) MS/MS spectra of synthetic M15-MP and reference. (D) Under collision-induced dissociation (CID) conditions, M15-MP undergoes mainly retro-Diels-Alder (RDA) fragmentation.

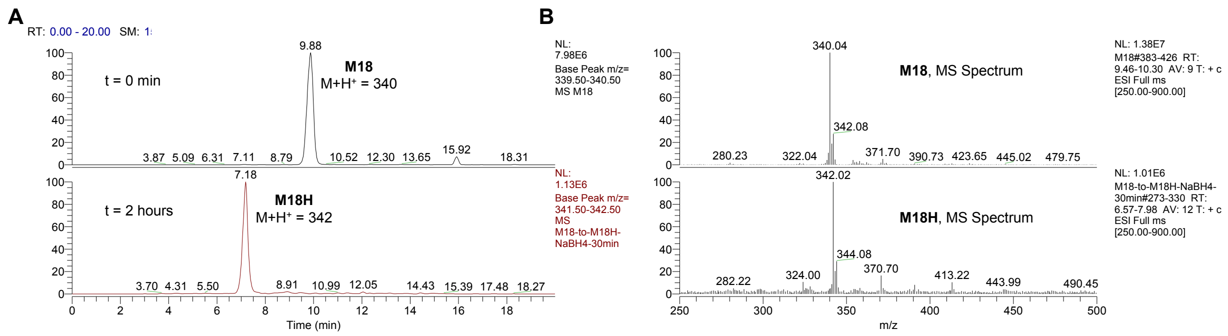

Supplementary Figure 3. Chemical preparation of M18H reference from M18 stock solution. (A) LC-MS/MS studies of the reductive conversion (all the diastereomers were separated on UPLC in Figure 2). (B) MS spectra of M18 and M18H.

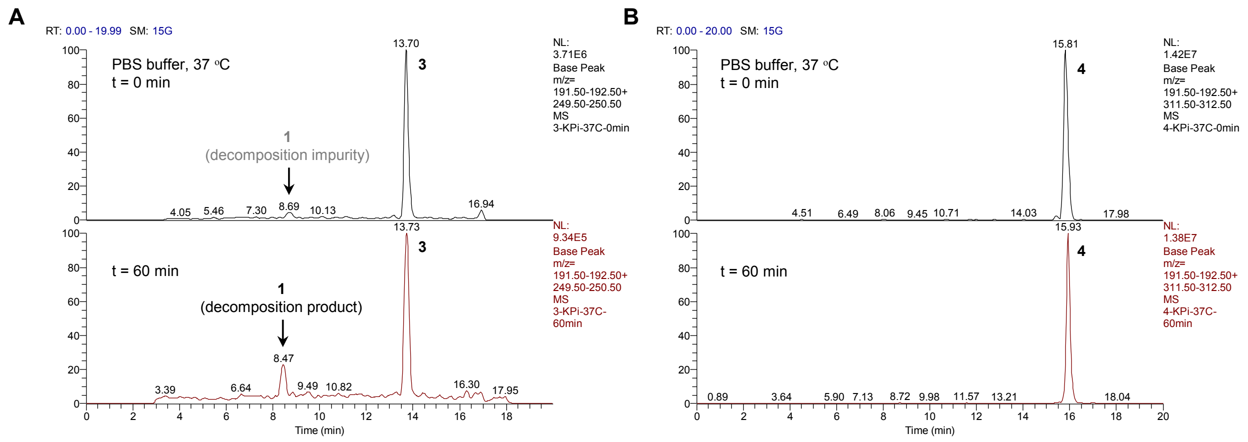

Supplementary Figure 4. Chemical stability test of model donor 3 (A) and 4 (B) in PBS buffer. Model donor **3** has shown some minor degradation product after 60 min incubation at 37 ^{o}C while model donor **4** has shown to be very stable.

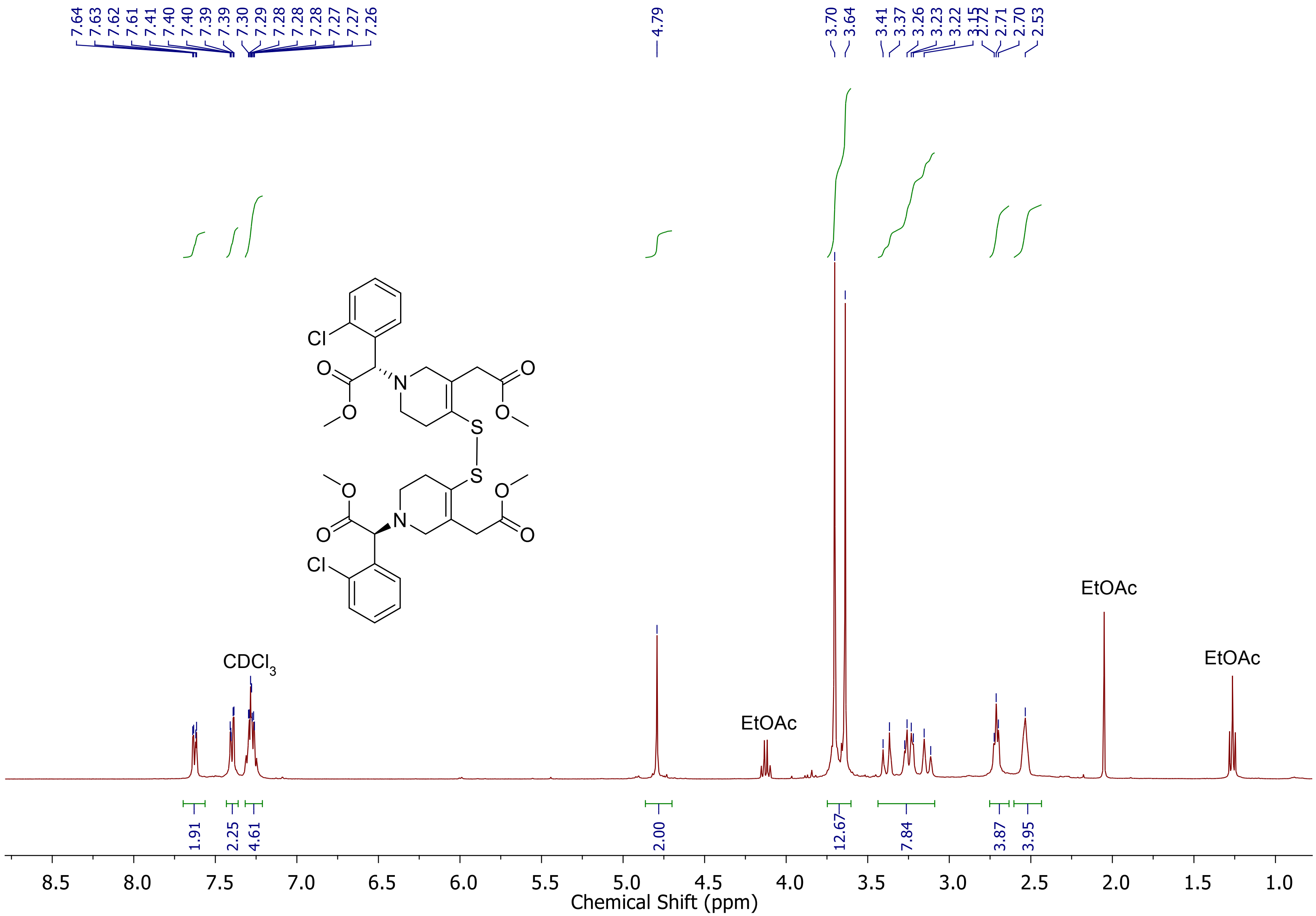

Supplementary Figure 5. ^{1}H-NMR ($CDCl_3$, 400 MHz) **M15-OMe-DS**.

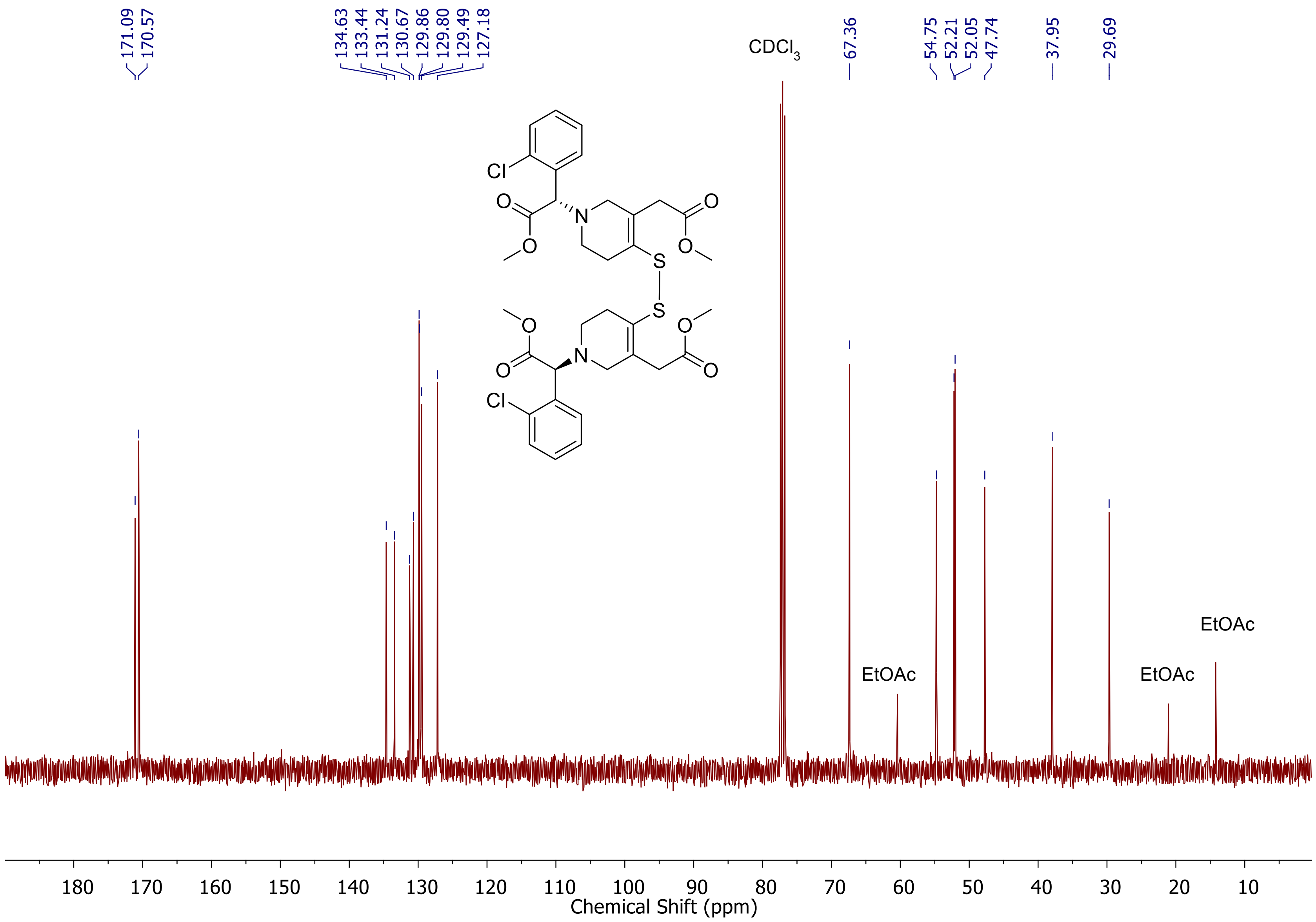

Supplementary Figure 6. ^{13}C-NMR ($CDCl_3$, 100 MHz) **M15-OMe-DS**.

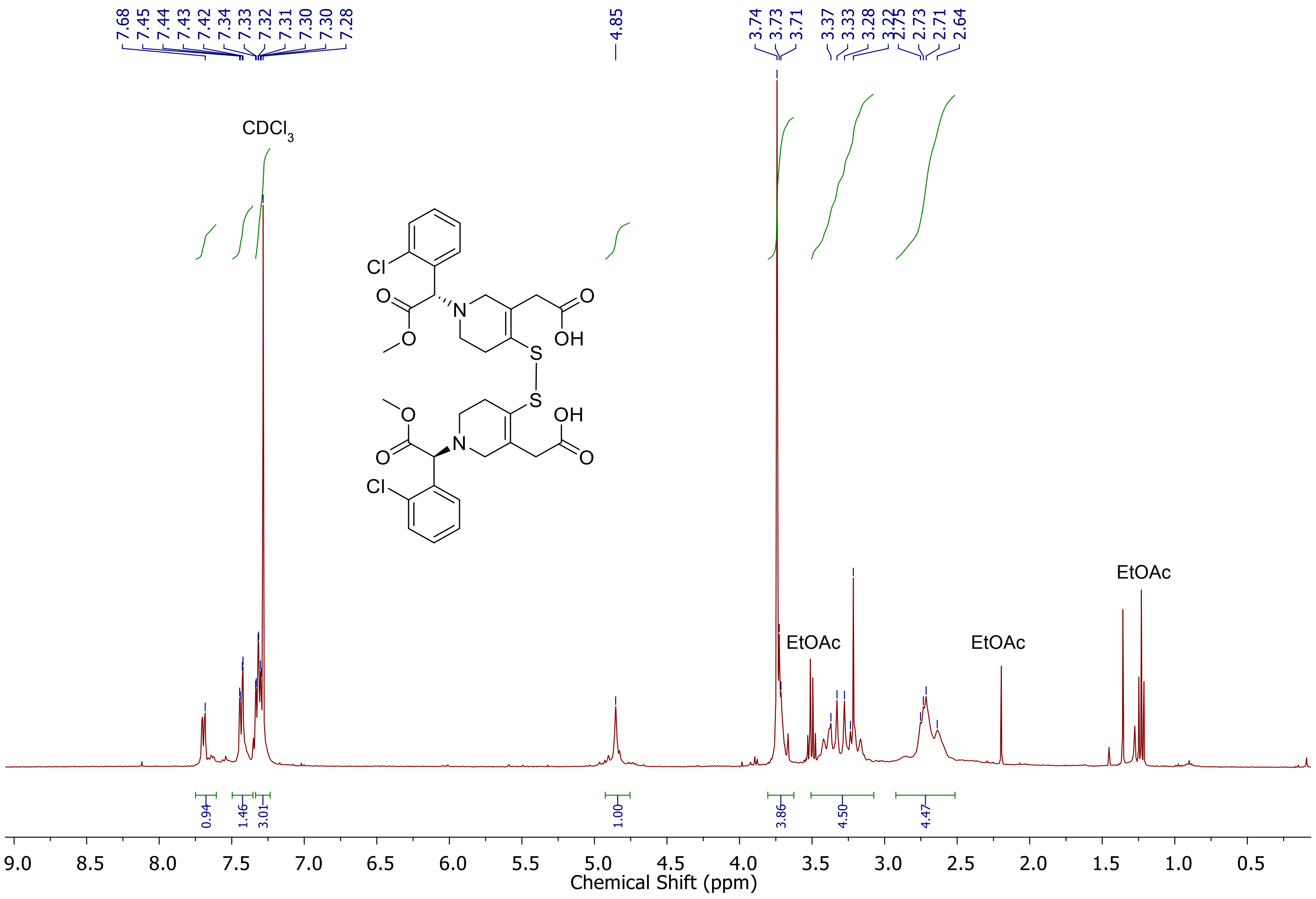

Supplementary Figure 7. ^{1}H-NMR ($CDCl_3$, 400 MHz) **M15-DS**.

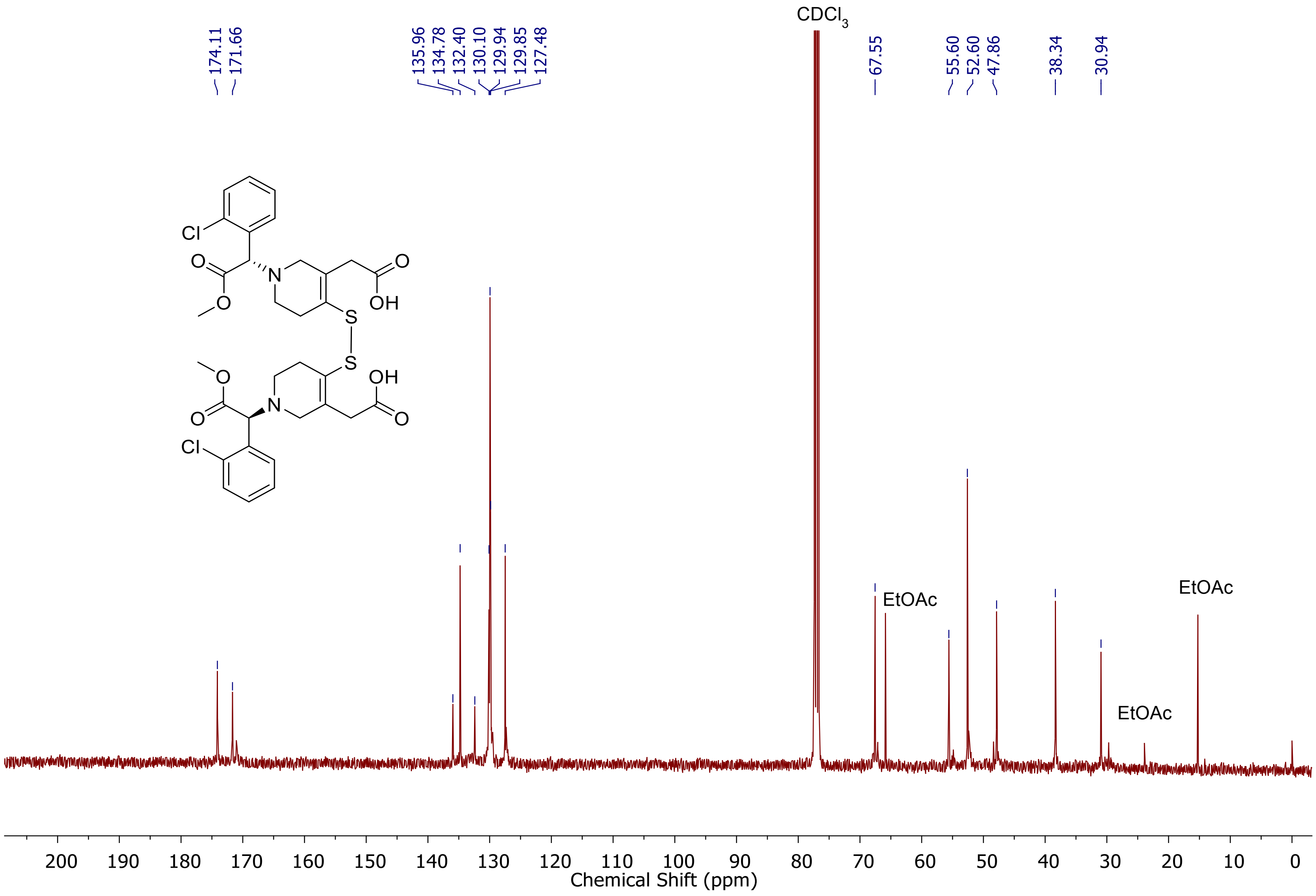

Supplementary Figure 8. ^{13}C-NMR ($CDCl_3$, 100 MHz) **M15-DS**.

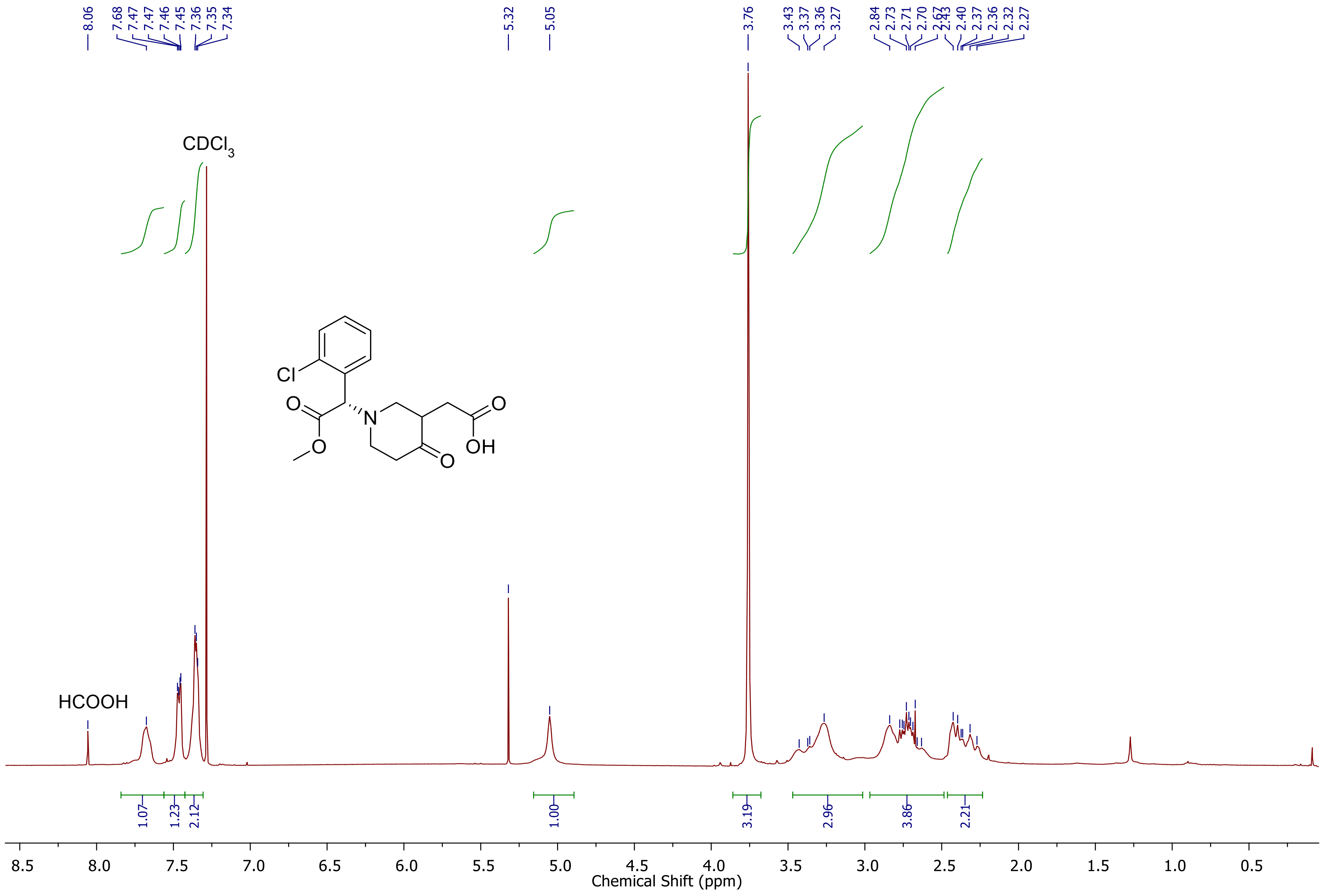

Supplementary Figure 9. ^{1}H-NMR ($CDCl_3$, 400 MHz) **M18**.

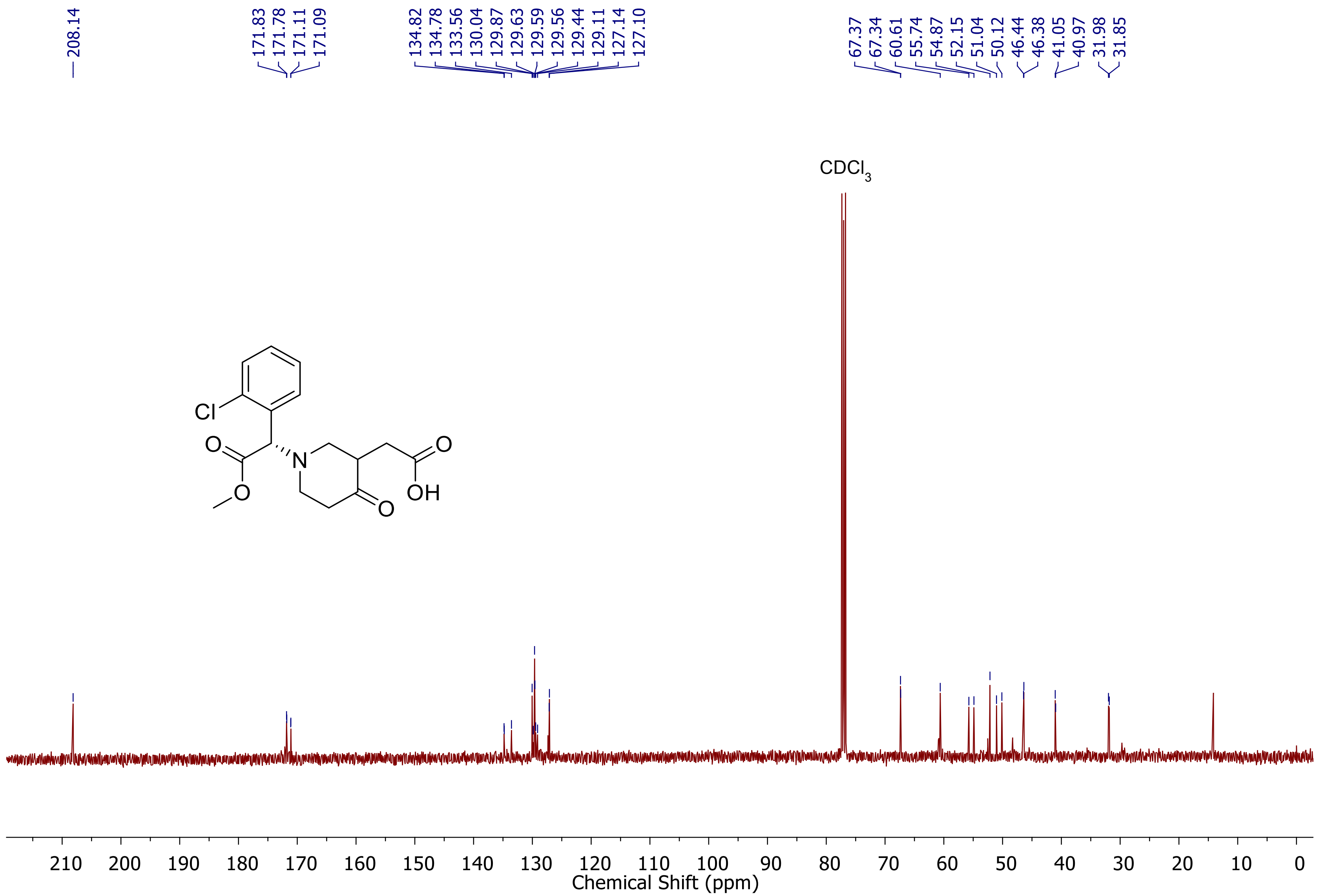

Supplementary Figure 10. ^{13}C-NMR ($CDCl_3$, 100 MHz) **M18**.

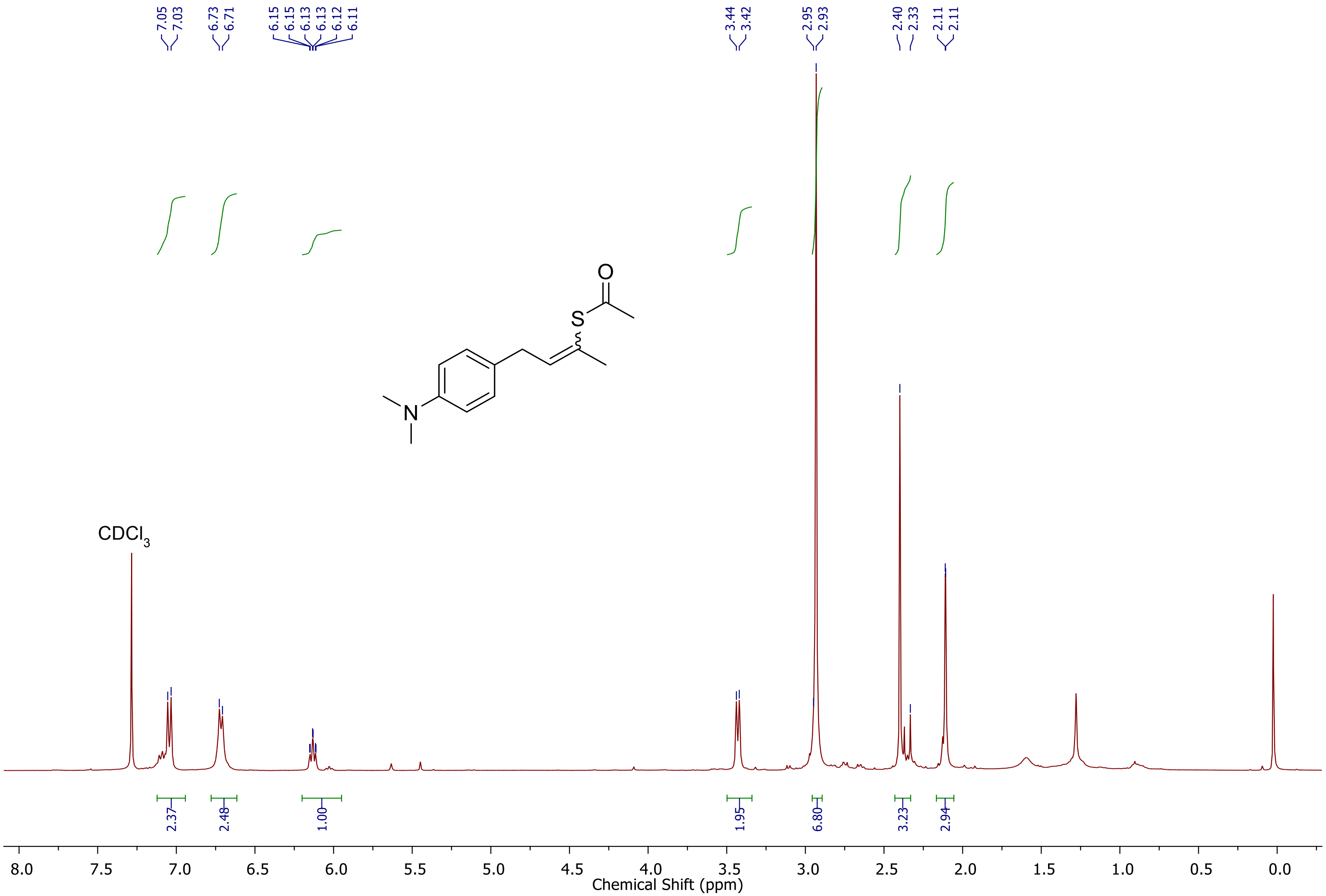

Supplementary Figure 11. ^{1}H-NMR ($CDCl_3$, 400 MHz) **3**.

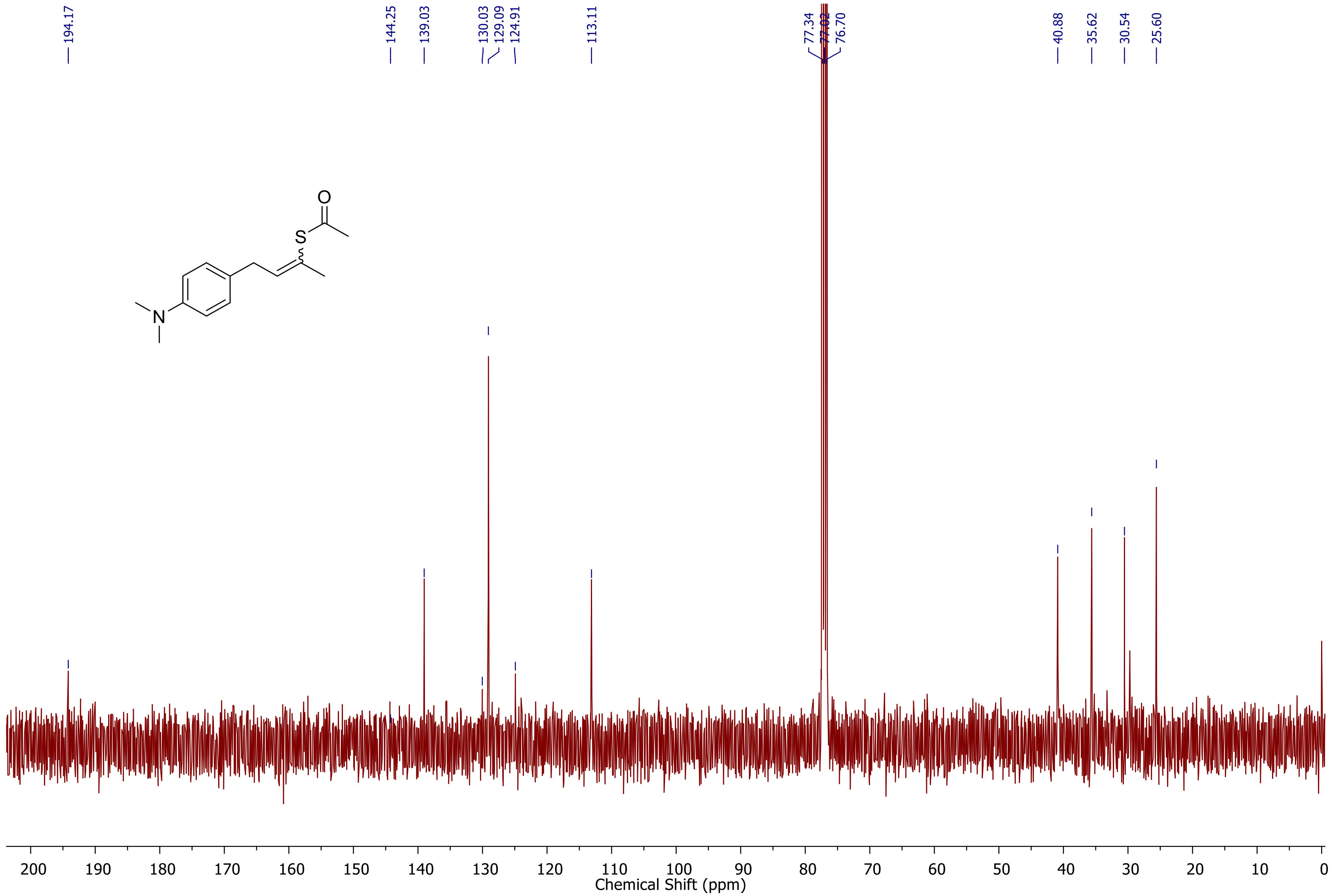

Supplementary Figure 12. ^{13}C-NMR ($CDCl_3$, 100 MHz) **3**.

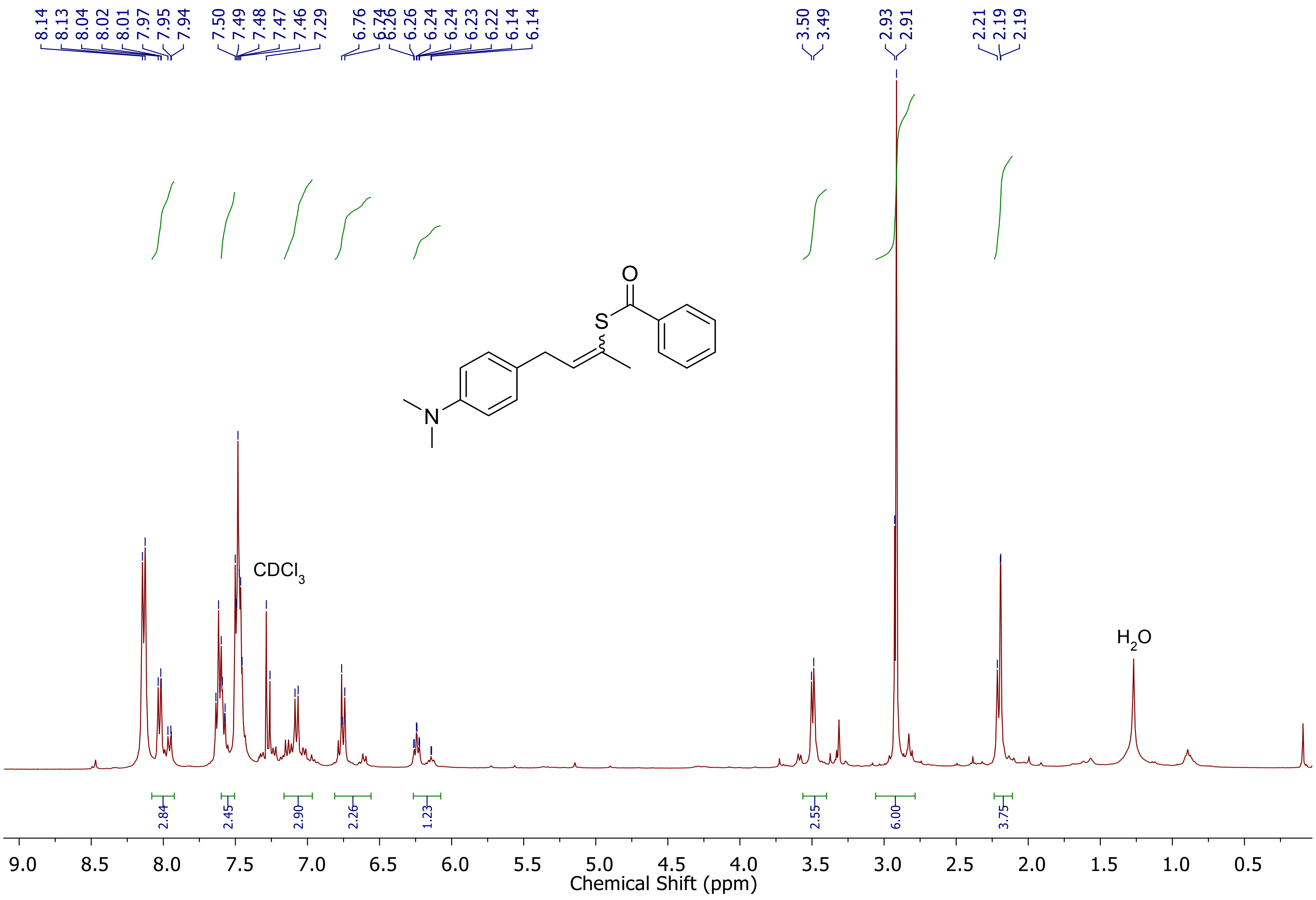

Supplementary Figure 13. ^{1}H-NMR ($CDCl_3$, 400 MHz) **4**.

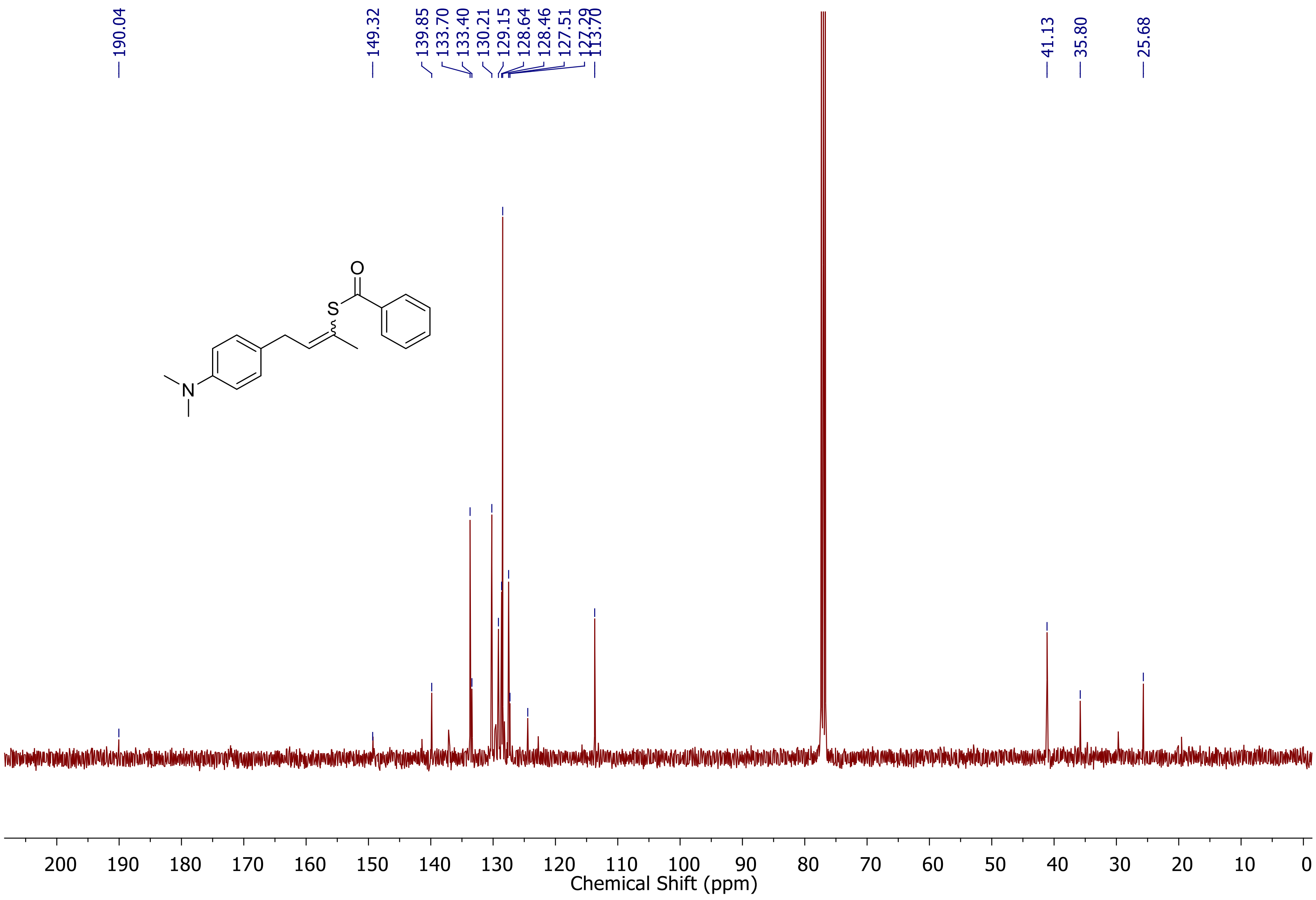

Supplementary Figure 14. ^{13}C-NMR ($CDCl_3$, 100 MHz) **4**.

Supplementary Methods

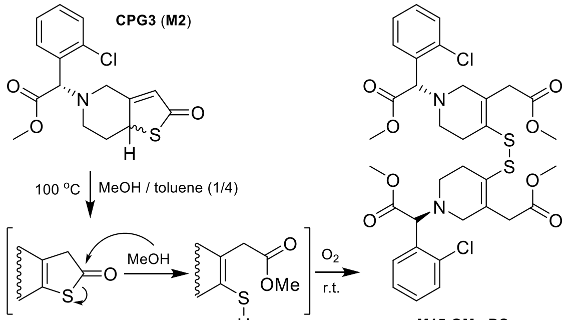

Disulfide of endo metabolite (M15) methyl ester (M15-OMe-DS): CPG3 was synthesized from chirally pure Methyl 2-chloro-D-mandelate (TCI America) according to literature procedures[1] and converted to HCl salt using 4 M HCl in diethyl ether. CPG3 (HCl salt, 200 mg, 0.53 mmol) was dissolved in a mixture of methanol and toluene (1:4, v/v), and the mixture was refluxed at 100 °C for 3 hours. The solvent was evaporated and the crude was purified by flash column chromatography (ethyl acetate/hexanes = 1/2) to afford M15-OMe-DS (87 mg, 44%) as yellow oil. ^{1}H NMR (400 MHz, $CDCl_3$) (**Supplementary Fig. 5**) δ 7.61-7.64 (m, 2 H), 7.39-7.41 (m, 2 H), 7.27-7.30 (m, 4 H), 4.79 (s, 2 H), 3.64-3.70 (m, 12 H), 3.15-3.41 (m, 8H), 2.70-2.71 (m, 4H), 2.51-2.54 (m, 4H); ^{13}C NMR (100 MHz, $CDCl_3$) (**Supplementary Fig. 6**) δ 171.1, 170.6, 134.6, 133.4, 131.2, 130.7, 129.9, 129.8, 129.5, 127.2, 67.36, 54.8, 52.2, 52.1, 47.7, 37.9, 29.7. HRMS m/z calcd for $C_{34}H_{39}Cl_2N_2O_8S_2$ $[M+H]^+$ 737.1519, found 737.1519. The endo structure is supported by NMR studies (**Supplementary Fig. 1**).

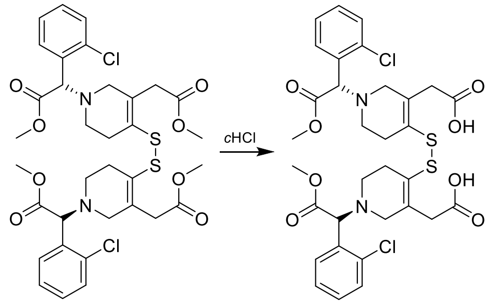

Disulfide of M15 (M15-DS): M15-OMe-DS (100 mg, 0.14 mmol) was stirred in concentrated HCl (1 mL) for 12 hours at room temperature. Reaction was monitored by LC-MS and evaporated to dryness and lyophilized to obtain crude as a pale yellow solid. The crude was purified by using semi-preparative LC to yield M15-DS (35 mg, 36%) as a light yellow powder. ^{1}H NMR (400 MHz, $CDCl_3$) (**Supplementary Fig. 7**) δ; 7.69 (dd, J = 7.4, 2.0 Hz, 2H), 7.43 (dd, J = 7.5, 1.8 Hz, 2H), 7.36-7.22 (m, 4H), 4.85 (s, 2H), 3.82-3.65 (m, 6H), 3.53-3.08 (m, 8H), 2.71 (dd, J = 27.0, 19.2 Hz, 8H); ^{13}C NMR (100 MHz, $CDCl_3$) (**Supplementary Fig. 8**) δ 174.1, 171.7, 136.0, 134.8, 132.4, 130.1, 129.9, 129.9, 127.5, 67.6, 55.6, 52.6, 47.9, 38.3, 30.9. HRMS m/z calcd for $C_{32}H_{35}Cl_2N_2O_8S_2$ $[M+H]^+$ 709.1206, found 709.1210.

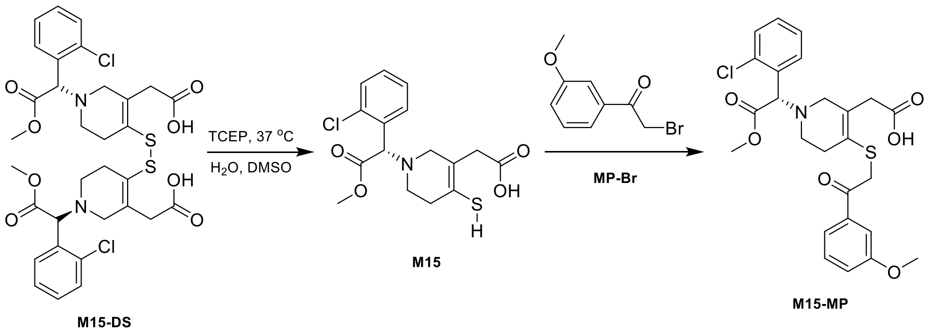

M15-MP: M15-DS (10 mg, 0.014 mmol) and TCEP (40 mg, 0.14 mmol) were dissolved in 1 mL of H_2O/DMSO mixture (1:1, v/v) and stirred at 37 °C. After 1 hour, 3'-methoxyphenacyl bromide (MP-Br, 32 mg, 0.14 mmol) was added, and the reaction mixture was stirred for another 1 hour. Methanol (1 mL) and saturated $NaHCO_3$ (1 mL) were added, and the mixture was purified by semi-preparative LC followed by lyophilization to afford M15-MP. LC-MS/MS studies have confirmed the synthetic M15-MP to be identical to the clinical reference purchased from Toronto Research Chemicals (**Supplementary Fig. 2**).

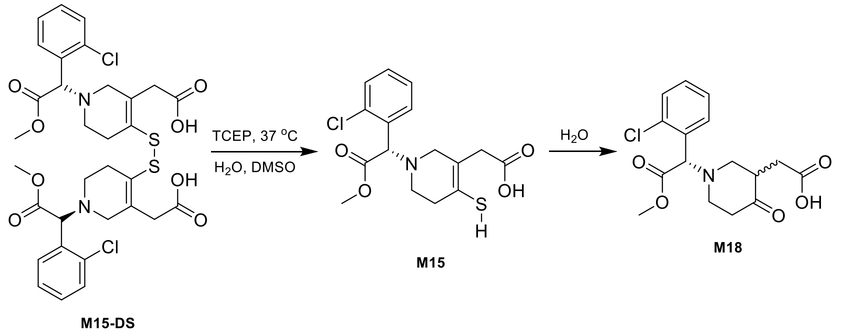

Metabolite M18 Reference: M15-DS (50 mg, 0.07 mmol) was dissolved in 1 mL of H_2O/DMSO mixture (1:1, v/v) and stirred at room temperature for 2 hours. The reaction mixture was purified by semi-preparative LC followed by lyophilization to yield M18 as a light yellow powder (11.5 mg, 24%). ^1H NMR (400 MHz, $CDCl_3$) (**Supplementary Fig. 9**) δ; 7.68-7.65 (m, 1H), 7.47-7.45 (m, 1H), 7.36-7.34 (m, 2H), 5.05 (s, 1H), 3.76 (s, 3H), 3.43-3.32 (m, 3 H), 2.84-2.67 (m, 4H), 2.43-2.32 (m, 2H). ^{13}C NMR (100 MHz, $CDCl_3$) (**Supplementary Fig. 10**) δ; 208.1, 171.8, 171.7, 171.1, 171.0, 134.8, 134.7, 133.6, 130.0, 129.9, 129.6, 126.5, 129.5, 129.4, 129.1, 127.1, 127.1, 67.4, 67.3, 60.6, 55.7, 54.9, 52.2, 51.0, 46.4, 46.3, 41.0, 40.9, 31.9, 31.8. HRMS m/z calcd for $C_{16}H_{19}ClNO_5$ $[M+H]^+$ 340.0946, found 340.0941.

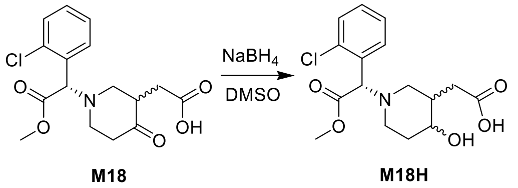

Metabolite M18H Reference: In a 0.5 mL of Eppendorf centrifuge tube was added 1 mg of $NaBH_4$ followed by 100 μL of 10 mM stock solution of M18. The tube was put a mixer for 2 hours at room temperature. LC-MS/MS showed full reductive conversion (**Supplementary Fig. 3**).

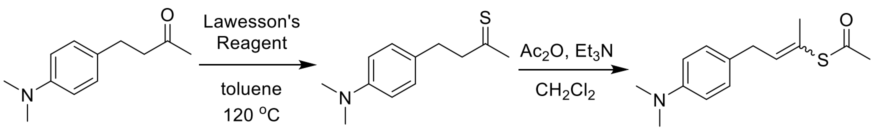

Model donor 3. Model compound **1** was synthesized according to literature procedures.[2] **1** (100 mg, 0.52 mmol) was dissolved in toluene (20 mL), and Lawesson's reagent was added (254 mg, 0.63 mmol). The reaction mixture was refluxed for 2 hours at 120 °C. After cooled down, the solvent was evaporated, and the residuals were passed through a silica gel column (ethyl acetate/hexanes = 1/4) to separate most of the side products for obtaining a crude product of **2**. The crude product was dissolved in dichloromethane (DCM, 2 mL), and trimethylamine (Et_3N, 145 μL, 1.04 mmol) and acetic anhydride (Ac_2O, 98 μL, 1.04 mmol) were added. After stirred at room temperature for 4 hours, the reaction mixture was washed with brine (10 mL) and water (10 mL) and extracted with ethyl acetate (3 x 20 mL). The organic layers were combined, dried over Na_2SO_4, concentrated, and purified by preparative TLC (ethyl acetate/hexanes = 1:8) to yield **3** (22 mg, 15%, two steps) as a yellow solid. ^{1}H NMR (400 MHz, $CDCl_3$) (**Supplementary Fig. 11**) δ 7.04 (d, J = 8.5 Hz, 2H), 6.72 (d, J = 8.0 Hz, 2H), 6.13 (t, J = 7.4, Hz, 1H), 3.43 (d, J = 7.2 Hz, 2H), 2.94 (s, 6H), 2.4 (s, 3H), 2.11 (s, 3H). ^{13}C NMR (100 MHz, $CDCl_3$) (**Supplementary Fig. 12**) δ 194.2, 144.3, 139.0, 130.0, 129.1, 124.9, 113.1, 40.9, 35.6, 30.5, 25.6. HRMS m/z calcd for $C_{14}H_{19}NOS$ $[M+H]^+$ 250.1260, found 250.1259. The chemical stability of model donor 3 is shown in **Supplementary Fig. 4 (A)**.

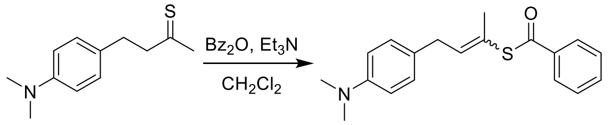

Model donor 4. **1** (100 mg, 0.52 mmol) was dissolved in toluene (20 mL), and Lawesson's reagent was added (254 mg, 0.63 mmol). The reaction mixture was refluxed for 2 hours at 120 °C. After cooled down, the solvent was evaporated, and the residuals were passed through a silica gel column (ethyl acetate/hexanes = 1/4) to separate most of the side products for obtaining a crude product of **2**. The crude product was dissolved in dichloromethane (DCM, 2 mL), and trimethylamine (Et_3N, 145 μL, 1.04 mmol) and benzoic anhydride (Bz_2O, 234 mg, 1.04 mmol) were added. After stirred at room temperature for 4 hours, the reaction mixture was washed with brine (10 mL) and water (10 mL) and extracted with ethyl acetate (3 x 20 mL). The organic layers were combined, dried over Na_2SO_4, concentrated, and purified by preparative TLC (ethyl acetate/hexanes = 1:8) to yield **4** (35 mg, 22%, two steps) as a yellow solid. ^{1}H NMR (400 MHz, $CDCl_3$) (**Supplementary Fig. 13**) δ 8.09-7.94 (m, 2H), 7.67-7.56 (m, 3H), 7.14-6.98 (m, 2H), 6.79-6.66 (m, 2H), 6.30-6.20 (m, 1H), 3.50 (d, J = 7.3 Hz, 2H), 2.94 (m, 6H), 2.20 (m, 3H). ^{13}C NMR (100 MHz, $CDCl_3$) (**Supplementary Fig. 14**) δ 190.0, 149.3, 139.9, 133.7, 133.4, 130.2, 129.2, 128.6, 128.5, 127.5, 127.3, 113.7, 41.1, 35.8, 25.7. HRMS m/z calcd for $C_{14}H_{19}NOS$ $[M+H]^+$ 312.1422, found 312.1416. The chemical stability of model donor 3 is shown in **Supplementary Fig. 4 (B)**.

Supplementary References

1. Shan, J. et al. Overcoming clopidogrel resistance: discovery of vicagrel as a highly potent and orally bioavailable antiplatelet agent. *J. Med. Chem.* **55**, 3342-3352 (2012).
2. Dhuru, S. et al. Novel diarylheptanoids as inhibitors of TNF-α production. *Bioorg. Med. Chem. Lett.* **21**, 3784-3787 (2011).
